# Antimicrobial Activity, Genetic Diversity and Safety Assessment of Lactic Acid Bacteria Isolated from European Hakes (*Merluccius merluccius*, L.) Caught in the Northeast Atlantic Ocean

**DOI:** 10.3390/antibiotics14050469

**Published:** 2025-05-06

**Authors:** Lara Díaz-Formoso, Diogo Contente, Javier Feito, Belén Orgaz, Pablo E. Hernández, Juan Borrero, Estefanía Muñoz-Atienza, Luis M. Cintas

**Affiliations:** 1 Grupo de Seguridad y Calidad de los Alimentos por Bacterias Lácticas, Bacteriocinas y Probióticos (Grupo SEGABALBP), Sección Departamental de Nutrición y Ciencia de los Alimentos (Nutrición, Bromatología, Higiene y Seguridad Alimentaria) (SD-NUTRyCIAL), Facultad de Veterinaria, Universidad Complutense de Madrid, Avda. Puerta de Hierro, s/n, 28040 Madrid, Spain; lardia01@ucm.es (L.D.-F.); diogodas@ucm.es (D.C.); ehernan@vet.ucm.es (P.E.H.); jborrero@ucm.es (J.B.); lcintas@vet.ucm.es (L.M.C.); 2 Sección Departamental de Farmacia Galénica y Tecnología Alimentaria (SD-FARMATEC), Facultad de Veterinaria, Universidad Complutense de Madrid, Avda. Puerta de Hierro, s/n, 28040 Madrid, Spain; belen@vet.ucm.es

**Keywords:** European hakes (*Merluccius merluccius*, L.), aquaculture, probiotics, Lactic Acid Bacteria, antibiotic resistance

## Abstract

**Background/Objectives:** The overuse and misuse of antibiotics has contributed significatively to the growing problem of the emergence and spread of antibiotic resistance genes among bacteria, posing a serious global challenge to the treatment of bacterial infectious diseases. For these reasons, there is a current and growing interest in the development of effective alternative or complementary strategies to antibiotic therapy for the prevention of fish diseases, which are mainly based on the use of probiotics—in particular, those belonging to the Lactic Acid Bacteria (LAB) group. In this context, the aim of the present study was to characterise, evaluate the genetic diversity and assess the safety of candidate probiotic LAB strains for aquaculture isolated from faeces and intestines of European hakes (*Merluccius merluccius*, L.) caught in the Northeast Atlantic Ocean (Ireland). **Methods:** The direct antimicrobial activity of the LAB isolates was tested by the Stab-On-Agar method against key ichthyopathogens. Subsequently, their taxonomic classification and genetic diversity were determined by *16SrDNA* sequencing and Enterobacterial Repetitive Intergenic Consensus-PCR (ERIC-PCR), respectively. To ensure the in vitro safety of the LAB isolates, their biofilm-forming ability was assessed by a microtiter plate assay; their sensitivity to major antibiotics used in aquaculture, human and veterinary medicine by a broth microdilution method and their haemolytic and gelatinase activity by microbiological assays. **Results:** All LAB isolates were biofilm producers and susceptible to chloramphenicol, oxytetracycline, flumequine and amoxicillin. A total of 30 isolates (85.7%) were resistant to at least one of the tested antibiotics. None of the 35 LAB isolates showed haemolytic or proteolytic activity. **Conclusions:** Among the isolated strains, five LAB strains exhibiting the highest antimicrobial activity against aquaculture-relevant ichthyopathogens, taxonomically identified as *Streptococcus salivarius*, *Enterococcus avium* and *Latilactobacillus sakei*, were selected for further characterisation as potential probiotic candidates to promote sustainable aquaculture. To our knowledge, this is the first study to report that hake intestines and faeces represent viable ecological niches for the isolation of LAB strains with antimicrobial activity.

## 1. Introduction

Aquaculture is currently emerging as a successful strategy to maintain global food security for fish and other fishery products in both industrialised and developing countries [1]. Since 9.8 billion people are expected to populate the planet by 2050, the aquaculture industry, like other food-producing sectors, must sustainably maintain its production growth [2,3]. Aquaculture produced an estimated 94 million tonnes of aquatic animals, surpassing capture fisheries for the first time [3]. In this regard, one of the main problems facing modern aquaculture is to provide a sufficient and constant supply of products with adequate nutritional, hygienic and sanitary quality and safety, as well as the prevention and control of infectious fish diseases, mainly caused by bacteria, viruses, protozoa, helminths and fungi at the larval stages [4,5,6,7,8,9,10,11].

Traditionally, antibiotics have been used as a therapeutic and prophylactic treatment for bacterial ichthyopathologies. However, there is an increasing reluctance and restriction on their use, not only by health agencies but also by the fish farms and consumers, due to their detrimental effects on animal and human health, food safety and the environment [1,12]. In this respect, the excessive use of antibiotics in aquaculture has contributed to the growing and serious problem of the emergence and spread of transmissible bacterial resistance genes to (multiple) antibiotics, which is a major global problem for the treatment of infectious diseases of bacterial aetiology [13,14,15,16,17]. It should be noted that, although vaccination can be an effective strategy for the prevention of ichthyopathologies, its application in aquaculture is difficult due to the limited number of effective commercial vaccines, the variability of the degree and period of protection they offer, the problems of their application at the larval stages, both because of the immaturity of their immune system and the difficulty of their handling, the growth retardation that they can cause, the stress that their use can cause in fish and the alterations that they can cause in the quality of the product [18,19]. Moreover, there are currently no effective vaccines available for the treatment of many of the bacterial, viral and parasitic diseases affecting fish, so the only way to control them is by preventing infections through biosecurity measures and/or by using antimicrobial compounds (antibiotics, antivirals and antiparasitics) [20,21,22]. For all these reasons, there is currently a great interest in the development of novel and effective strategies for the prevention of bacterial ichthyopathologies as alternative or complementary strategies to antibiotherapy and vaccination, such as the use of probiotics, which are defined as live microorganisms that confer a health benefit to the host [17,23,24,25].

Lactic Acid Bacteria (LAB) are the safest and most frequently proposed bacteria for the development of probiotics for aquaculture due to their Qualified Presumption of Safety (QPS) status recognized by the European Food Safety Authority (EFSA) and because several strains are legally accepted for use as probiotics in humans and as zootechnical additives [26]. However, so far, the only strain authorized in the EU as a probiotic for aquaculture is *Pediococcus acidilactici* CNCM I-4622, due to its beneficial effect on the growth and functional development of fish and crustaceans. In addition, LAB have been evaluated as probiotics due to their antimicrobial properties, such as the production of organic acids and ribosomally synthetized antimicrobial peptides, referred to as bacteriocins [23,24,27]. The use of bacteriocin-producing LAB as probiotics and biocontrol agents in aquaculture could play a crucial role in improving animal health, productivity and product quality, while reducing the environmental impact [5,28,29,30,31].

The first step in this process is acquiring a collection of bacteriocinogenic LAB with suitable probiotic potential. It is also suggested that isolating bacteria from aquatic environments or host-related bacteria can enhance the adaptive capacity of native microbiota, improving their ability to colonize the gastrointestinal tract, mucous membranes and aquatic ecosystems [28,30,31,32,33,34,35,36,37,38]. According to this, the use of isolated and characterised LAB of fish origin could contribute to the development of a sustainable aquaculture in the One Health context, not only for the prevention and control of the most relevant ichthyopathologies but also to contribute to their health, immune status and to the productivity and economic profitability of aquaculture farms. Additionally, fish and fishery products are among the most traded food products worldwide, because they are considered an important source of proteins and minerals. Specifically, the European hake (*Merluccius merluccius*, L.) is one of the most popular and appreciated white fish, with a significant commercial impact on the fishing industry. Specifically, 62,500 tonnes of European hakes were consumed in Spain in 2021 [3,39]. In this context, the objective of this work was the characterization, genetic diversity evaluation and safety assessment of candidate probiotic LAB strains for aquaculture isolated from the faeces and intestines of European hakes (*Merluccius merluccius* L.) captured in the Northeast Atlantic Ocean (Ireland).

## 2. Results

### 2.1. Antimicrobial Activity and Taxonomic Identification of Bacteria Isolated from European Hakes

A total of 286 bacterial isolates from De Man, Rogosa and Sharpe (MRS) agar plates, which is an elective medium for the isolation of LAB, were selected, according to their different colony morphologies and including representatives from the eight hake samples for the evaluation of their antimicrobial activity. The pre-selection of isolates was made based on their antimicrobial activity spectrum. This involved selecting isolates that represented all the identified groups of antimicrobial activities. In this sense, both isolates with narrow and broad spectra of activity were selected. Conversely, those strains that did not show activity against any indicator were discarded. A total of 66 isolates were pre-selected for their taxonomic identification. The pre-selected isolates were taxonomically identified as *Staphylococcus* spp. (41%), *Lactococcus* spp. (32%), *Streptococcus* spp. (9%), *Enterococcus* spp. (9%), *Macrococcus* spp. (1.5%), *Bacillus* spp. (1.5%), *Lactobacillus* spp. (1.5%), *Leuconostoc* spp. (1.5%), *Aerococcus* spp. (1.5%) and *Rothia* spp. (1.5%). Bacterial isolates identified as LAB (*n* = 35) were selected for further safety assessment.

**Figure 1:**
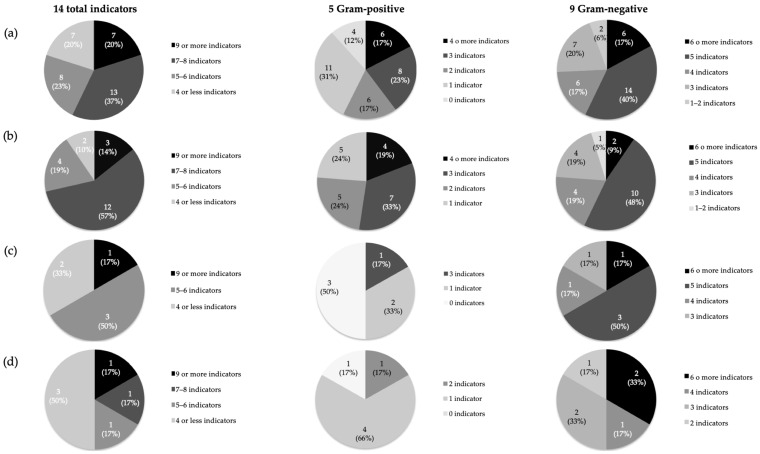
Distribution of the total 35 LAB isolates (**a**), 21 *Lc. garvieae* (**b**), 6 *St. salivarius* (**c**) and 6 *E. avium* (**d**) according to their direct antimicrobial activity against the 14 microorganisms used as indicators (5 Gram-positive and 9 Gram-negative) by a SOAT.

The results of the direct antimicrobial activity of the 35 LAB isolates are shown in Table S1. In brief, all isolates exhibited a broad antimicrobial spectrum, inhibiting the growth of at least three indicator strains and 57% inhibited the growth of at least half of the indicator microorganisms (Figure 1). Specifically, 23% of the LAB isolates inhibited five to six indicators, 37% inhibited seven to eight and 20% inhibited nine or more (Figure 1). The most sensitive indicators were the Gram-negative fish pathogens *Aeromona salmonicida* CLFP-23 and *Aeromona salmonicida* CECT4237, followed by *Streptococcus parauberis* LMG22252 and *Tenacibacullum maritimum* NCIMB2154, all of them reported as important fish pathogens [40,41,42]. In the case of *Lactococcus garvieae*, 71% inhibited at least half of the tested indicators, 52% inhibited at least three Gram-positive indicators and 57% inhibited at least five Gram-negative indicators. Moreover, 67% of *Streptococcus salivarius* inhibited at least five indicators, 50% inhibited three Gram-positive indicators and 67% inhibited more than five Gram-negative indicators. In addition, 34% of *Enterococcus avium* inhibited half of the indicators—specifically, 83% inhibited at least one Gram-positive indicator, and all of them (100%) inhibited at least two Gram-negative indicators (Figure 1).

### 2.2. Genetic Diversity Analysis by ERIC-PCR

**Figure 2:**
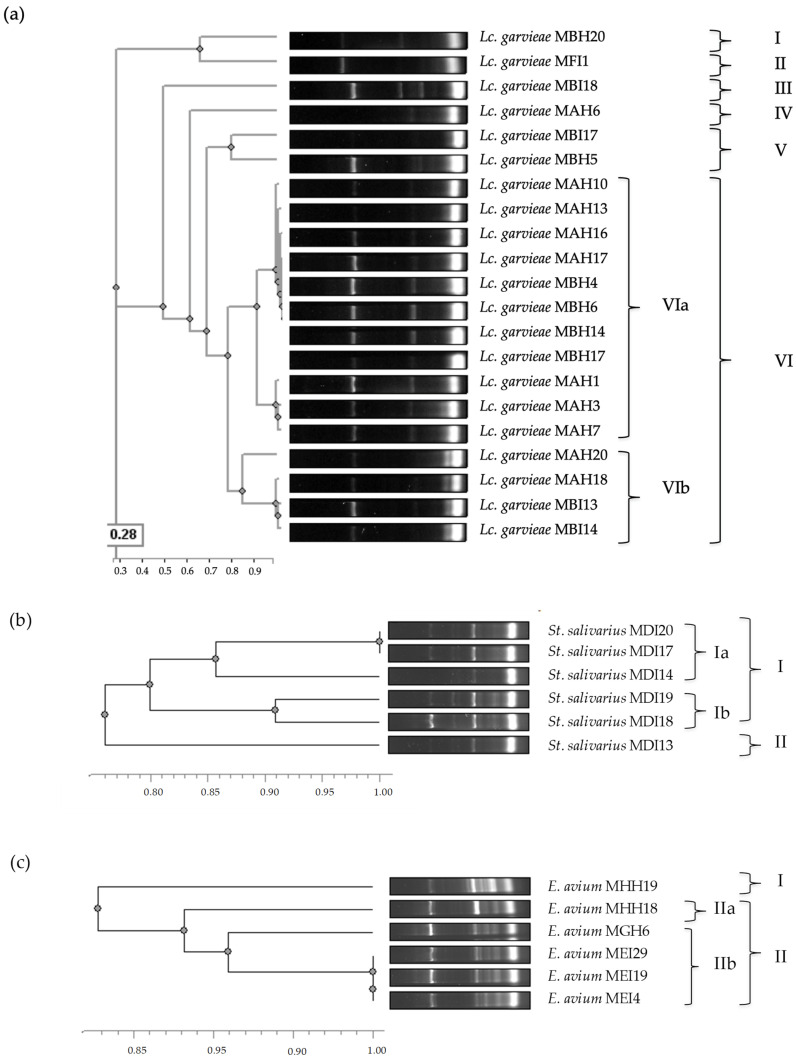
Phylogenetic relatedness of *Lc. garvieae* (**a**), *St. salivarius* (**b**) and *E. avium* (**c**) isolates from European hakes based on their ERIC-PCR patterns. Isolates with a similarity threshold ≥ 0.75 (**a**), ≥0.85 (**b**) and ≥0.90 (**c**) were considered to have closely related ERIC-PCR patterns.

Phylogenetic relatedness of the 35 LAB was determined by ERIC-PCR (Figure 2). *Lc. garvieae* isolates presented a high phylogenetic diversity, showing six different clusters (I-VI). *Lc. garvieae* isolates were identified in three different hakes (hakes A, B and F). Specifically, isolates from hake A were grouped into two different clusters (IV and VI), and isolates from hake B into four clusters (I, III, V and VI). In the case of *St. salivarius* isolated from hake D, they were highly similar (76%), detecting two ERIC-PCR patterns: cluster I, divided into Ia and Ib, and cluster II. On the other hand, *E. avium* isolates were found in three different hakes (hakes E, G and H) and presented a high phylogenetic relationship (83%). These isolates were grouped in cluster I and II, the latter divided into IIa and IIb. Interestingly, two isolates (*E. avium* MH19 and *E. avium* MH18) belonging to hake H were grouped into two different clusters, namely I and IIa, respectively. In addition, cluster IIb included the *E. avium* isolates from hakes E and G.

### 2.3. Biofilm Formation

**Figure 3:**
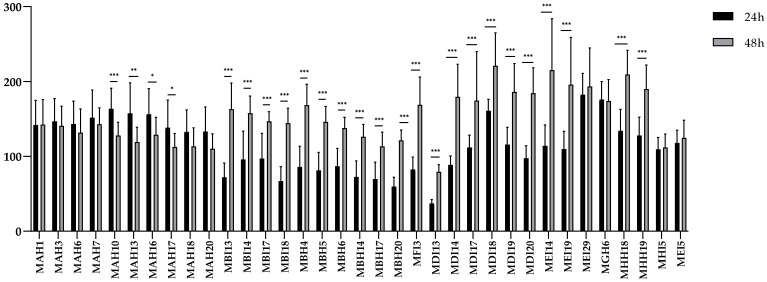
Time-dependent comparison of relative biofilm formation by LAB at 24 and 48 h incubation. The results are expressed as percentages relative to those of the reference strain (*S. aureus* ATCC 25923). Asterisks indicate significantly different levels between biofilm formation at 24 and 48 h (* *p* ≤ 0.05, ** *p* ≤ 0.01 and *** *p* ≤ 0.001).

All LAB isolates from hakes were biofilm producers (Figure 3), with *St. salivarius* MDI13 being the isolate that showed the lowest biofilm production. A comparison was made between biofilm formation at 24 and 48 h for the different isolates. Interestingly, 25 isolates (71.4%) showed significant differences, with 81% (*n* = 21) of them being higher biofilm producers at 48 h compared to 24 h. Specifically, 71.4% (*n* = 15) of the *Lc. garvieae* isolates showed significant differences, with 52.4% (*n* = 11) showing a significant increase in biofilm production at 48 h and only 19% (*n* = 4) showing a significant decrease at that time. *Lb. sakei* (*n* = 1) showed no significant differences in biofilm formation at both incubation times. With respect to *St. salivarius*, 100% (*n* = 6) showed a significant increase in biofilm formation at 48 h, and 66% of the *E. avium* isolates (*n* = 6) also showed a significant increase in biofilm formation at 48 h. However, *Leuconostoc carnosum* (*n* = 1) and *Lb. sakei* (*n* = 1) showed no significant differences at both incubation times.

### 2.4. LAB Susceptibility to Antibiotics

The susceptibility of the 35 LAB isolates to antibiotics recommended by the EFSA (i.e., ampicillin, vancomycin, gentamicin, kanamycin, streptomycin, erythromycin, clindamycin, tetracycline and chloramphenicol) [43] and to others used in aquaculture (i.e., florfenicol, oxytetracycline, flumequine, amoxicillin and trimethoprim-sulfamethoxazole) is shown in Table 1.

All the LAB isolates were susceptible to chloramphenicol, oxytetracycline, flumequine and amoxicillin. A total of 30 isolates (85.7%) were resistant to at least one of the tested antibiotics. The most frequently found resistances were to ampicillin (68.6%), kanamycin (62.8%), trimethoprim-sulfamethoxazole (62.8%) and clindamycin (62.8%), followed by streptomycin (48.6%), florfenicol (31.4%), gentamicin (11.4%), erythromycin (5.7%) and tetracycline (5.7%).

### 2.5. Haemolytic and Gelatinase Activity

None of the 35 LAB isolates showed haemolytic or proteolytic activity.

## 3. Discussion

In this work, 35 LAB isolates from faeces and intestines of European hakes, which showed antimicrobial activity against relevant Gram-positive and Gram-negative ichthyopathogens, were selected to assess their in vitro safety. LAB is the group of bacteria most frequently proposed as probiotics for aquaculture [23,24]. Previous studies have already described the great diversity of bacterial species, including LAB, found in fish or fishery products [14,38,46,47,48,49,50,51,52,53]. Specifically, some species and genera of LAB isolated in our study have also been identified in fish and fish products, such as *E. avium* isolated from the gut of rohu (*Labeo rohita*) and catla (*Catla catla*) [54], *Lc. garvieae* from Nile tilapia (*Oreochromis niloticus*), Japanese threadfin bream (*Nemipterus japonicus*) [55] and rainbow trout (*Oncorhynchus mykiss*, Walbaum) [56] and also *Streptococcus* spp. from fermented fish products [57]. LAB are the most studied group of microorganisms as probiotics, and there have been numerous reviews evaluating their application and results in aquaculture productions [31,58,59,60,61]. Microbial antagonism by LAB may be due to competition for nutrients and the production of organic acids and other antimicrobial metabolites, including ethanol, hydrogen peroxide and ribosomally synthesised peptides [24,62,63].

Previous studies have shown that food-producing animals are frequently colonised by biofilm-forming bacteria [64,65]. The increased biofilm formation at 48 h by LAB isolated from hakes has also been observed in LAB strains of other origins [66,67]. The formation of the three-dimensional biofilm architecture is a multi-step process involving adsorption, adhesion, microcolony formation, maturation and dispersion. In this respect, biofilm formation is considered a mechanism employed by probiotic bacteria to resist stress conditions, such as pH changes and lack of nutrients [68,69,70]. Interestingly, biofilm formation can induce a distinct physiological state in bacteria, often characterised by reduced growth and metabolic rates. However, several studies have shown that LAB within biofilms can not only sustain but sometimes enhance their production of antimicrobial compounds. For example, *Lactobacillus casei* and *Lactobacillus reuteri* have exhibited significant antimicrobial and antibiofilm activities under biofilm-forming conditions, particularly against antibiotic-resistant pathogens such as *Proteus mirabilis*. Biofilm formation may increase the tolerance of LAB to environmental stressors such as nutrient deprivation, improving their persistence and functional performance within the host. Therefore, rather than limiting the broad-spectrum antimicrobial potential of LAB, biofilm formation may actually enhance their resilience and effectiveness, making them even more suitable for probiotic applications [71,72].

In this work, a total of 30 LAB isolates (85.7%) were resistant to at least one of the antibiotics tested. It should be noted that *Lc. garvieae* is the species that showed more resistances to antibiotics (ampicillin, gentamicin, kanamycin, streptomycin, clindamycin, florfenicol and trimethoprim-sulfamethoxazole). The most common resistances found in *Lc. garvieae* were to clindamycin, trimethoprim-sulfamethoxazole and streptomycin, as similarly previously described for isolates of this species from other niches such as cultured rainbow trout and a rearing environment [73]. All *Lc. garvieae* tested in this study showed resistance to clindamycin, which is considered as an intrinsic trait of this species [44]. Indeed, intrinsic resistance to clindamycin was initially proposed as a selective criterion to distinguish between *Lc. garvieae* and *Lc. lactis* species [74]. However, the fact that *Lc. lactis* may possess the Macrolide–Lincosamide–Streptogramin B (MLSB) resistance gene *erm(B)* makes the resistance to this antibiotic not suitable for distinguishing between these two species [75]. Moreover, 100% and 80.9% of the *Lc. garvieae* isolates from hakes were resistant to trimethoprim-sulfamethoxazole and streptomycin, respectively, a finding also observed in previous studies [73,76]. These results suggest that streptomycin and trimethoprim-sulfamethoxazole resistances could be intrinsic characteristics of this species. Additionally, 90.4% of the *Lc. garvieae* isolates showed resistance to kanamycin, which has been previously reported [77]. Furthermore, 71.4% of the *Lc. garvieae* isolates showed resistance to ampicillin, which has been previously reported for *Lc. garvieae* isolated from both rainbow trout and cows [78,79]. Finally, 52.3% showed resistance to florfenicol, which agrees with recent studies [80]. *Lc. garvieae* is considered an emerging pathogen worldwide and of great importance not only for animal (fish and livestock) health but also for human health due to its high zoonotic potential and pathogenicity. Therefore, finding *Lc. garvieae* strains resistant to several antibiotics could hamper future treatments of this zoonosis with important implications for public health [81,82].

Regarding the *St. salivarius* isolates from hakes, two of them (33.3%) exhibited resistance to erythromycin, which is consistent with previous studies in which resistance to this antibiotic has been reported as a common trait of this species [83,84]. In addition, 33.3% and 16.6% of *St. salivarius* strains showed resistance to tetracycline and gentamicin, respectively. Resistance to these antibiotics among streptococcal species (e.g., *Streptococcus agalactiae*, *Streptococcus pneumoniae*, *Streptococcus pyogenes* and *Streptococcus dysgalactiae* subsp. *equisimilis*) is usually observed at high rates [85,86,87,88]. Previous studies have also detected resistance to other antibiotics, such as kanamycin and ampicillin, in streptococci [85,89,90,91]. In particular, in our study, half of the *St. salivarius* isolates (50%) were resistant to kanamycin and 66.6% to ampicillin.

With respect to the *E. avium* isolates from hakes, 66.6% showed resistance to ampicillin, which has been frequently reported for enterococci [92,93]. Resistance to β-lactam antibiotics is of particular concern, as these drugs, alone or in combination, are frequently used in the treatment of enterococcal infections. This type of resistance significantly limits therapeutic options and represents a challenge in the management of these infections [94,95,96,97]. In addition, one isolate of *E. avium* (16.6%) showed resistance to clindamycin, which has been previously described for enterococci isolated from shellfish and fish [98].

On the other hand, *Lt. carnosum* MHI5, the only isolate within this species found in hakes, showed resistance to trimethoprim-sulfamethoxazole, which has previously been suggested as an intrinsic antibiotic resistance in this species [99]. *Leuconostoc* are generally susceptible to β-lactam antibiotics, such as ampicillin [100]. However, *Lt. carnosum* species can show resistance to this antibiotic, as it was found in this study for *Lt. carnosum* MHI5, reaching a MIC of 4 µg/mL. This unusual behaviour highlights the need for constant monitoring of antimicrobial resistance [101].

In this study, 21 LAB isolates from hakes (60%) showed multidrug resistance (MDR), which is defined as the acquired resistance of a microorganism to at least one antibiotic in three or more antimicrobial categories [102]. Regarding this, 85,7% of the *Lc. garvieae* isolates were resistant to antibiotics belonging to the categories of β-lactams (ampicillin); aminoglycosides (gentamicin, kanamycin and/or streptomycin); amphenicols (florfenicol) and/or folate pathway antagonists (trimethoprim-sulfamethoxazole), and 50% of *St. salivarius* were resistant to β-lactams (ampicillin), aminoglycosides (kanamycin), macrolides (erythromycin) and/or tetracycline. The occurrence of MDR may be due to the acquisition of mobile genetic elements (MGEs), such as bacteriophages, plasmids and integrative and conjugative elements [103]. In recent years, the identification of antibiotic resistance in LAB has attracted considerable interest, as they may act as reservoirs of antibiotic resistance genes that could subsequently be transferred to commensal or pathogenic bacteria in animals and even humans [104,105,106,107]. The present findings highlight a concerning trend, with 60% of the isolates exhibiting MDR, underscoring the risk of horizontal gene transfer in aquatic environments and contributing to what has been described as a silent pandemic. The widespread presence of antibiotics in these ecosystems not only disrupts the native microbial communities but also promotes the dissemination of resistance genes. Although various strategies have been developed to remove antibiotics from aquatic environments, many are limited by high operational costs and the potential generation of secondary pollutants [108,109].

Only five strains (14,28%)—namely, *St. salivarius* MDI13, *St. salivarius* MDI20, *E. avium* MEI4, *E. avium* MGH6 and *Lb. sakei* MEI5—did not show resistance to any of the 15 antibiotics tested in this work. Regarding the latter, *Lb. sakei* species has previously been described as a possible probiotic for fish due to an enhanced humoral and cellular immune response [110].

The absence of haemolytic activity is considered as a safety trait for potential probiotic candidates. The ability to produce haemolysins is a characteristic of some pathogenic bacteria that can degrade local tissues, converting them into nutrients for the bacteria, increasing the severity of infections [111]. Moreover, the absence of gelatinase production makes these isolates safer for the host. Gelatinase is an extracellular metalloendopeptidase capable of degrading substrates in host tissues, thereby enhancing bacterial migration and diffusion through damaged tissues [112]. Based on these findings, five strains (*Streptococcus salivarius* MDI13 and MDI20, *Enterococcus avium* MEI4 and MGH6 and *Lactobacillus sakei* MEI5) were selected primarily based on safety criteria. These were the only strains that did not exhibit antibiotic resistance, haemolytic activity or gelatinase production. In addition to fulfilling these essential safety requirements, they also demonstrated strong antimicrobial activity and biofilm-forming capacity. This combination of safety and functional properties supports their selection as the most promising probiotic candidates. Future studies will aim to further assess their safety by analysing the virulence factors, antibiotic resistance genes and mobile genetic elements, as well as through complementary in vivo testing.

## 4. Materials and Methods

### 4.1. Study Area, Sample Collection and Bacterial Isolation

**Figure 4:**
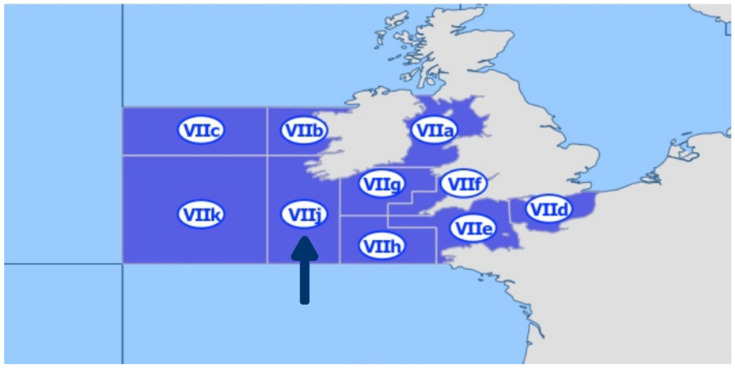
Map showing fishing area 27 (Atlantic, Northeast) according to the FAO, including the following divisions: VIIa (Irish Sea), VIIb (West of Ireland), VIIc (Porcupine Bank), VIId (Eastern English Channel), VIIe (Western English Channel), VIIf (Bristol Channel), VIIg (Celtic Sea North), VIIh (Celtic Sea South), VIIj (Southwest of Ireland/East) and VIIk (Southwest of Ireland—West). The arrow shows sub-area 27.VIIj where European hakes were caught. Source: https://fish-commercial-names.ec.europa.eu/fish-names/fishing-areas_en (accessed on 2 January 2025) [113].

Four European hake specimens from the Northeast Atlantic Ocean (Southwest of Ireland), specifically from the sub-area 27.VIIj (Figure 4), obtained during two consecutive years (June 2021 and June 2022) were sampled for this work [113].

**Figure 5:**
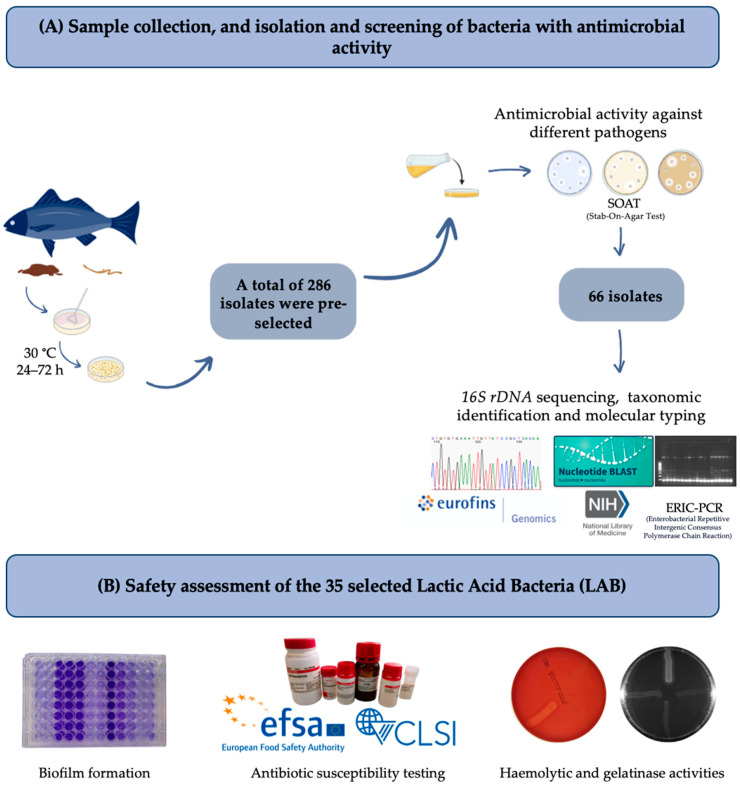
Schematic representation of the screening of bacteria isolated from European hakes. (**A**) Sample collection, isolation and screening of bacteria with antimicrobial activity. (**B**) Safety assessment of 35 LAB. This figure was created with BioRender.com.

For each sampling (June 2021 and 2022), four non-eviscerated commercial European hakes, provided by a Galician skipper dedicated to professional fishing, were used for bacteria isolation, making up a total of eight hake samples. For each hake, one gram of faeces was extracted, serially diluted in sterile peptone water (Oxoid Ltd., Basingstoke, UK) and spread over De Man, Rogosa and Sharpe (MRS, Oxoid, Basingstoke, UK) agar (1.5%, *w*/*v*, Scharlau, Barcelona, Spain) plates. In the case of the hake intestines, each sample was washed with phosphate-buffered saline (PBS; Oxoid Ltd., Basingstoke, UK) and homogenised in a stomacher (Seward, NY, USA) with sterile peptone water and then serially diluted and poured onto MRS agar plates (1.5%, *w*/*v*). The plates were incubated at 30 °C for 24–72 h in aerobiosis and anaerobiosis. A total of 286 isolates with different morphology, including representatives from the 8 hake samples, were selected to evaluate their antimicrobial activity. The schematic view of the screening used in this study is illustrated in Figure 5.

### 4.2. Screening of Bacteria with Antimicrobial Activity Against Ichthyopathogens

To assess the direct antimicrobial activity of the isolates and select those of great interest, a Stab On Agar Test (SOAT) was carried out against different ichthyopathogens of relevance to aquaculture (e.g., *A. salmonicida* CLFP-23, *A. salmonicida* CECT4237, *Aeromonas hydrophila* CECT839, *A. hydrophila* CECT5734, *Edwarsiella tarda* CECT886, *Lactococcus garvieae* CF00021, *Lc. garvieae* CLG4, *Listonella anguillarum* CECT4344, *St. parauberis* LMG22252, *T. maritimum* NCIMB2154, *T. maritimum* CECT1161 and *Yersinia ruckeri* LMG3279) and against other microorganisms of importance for humans (*Listeria monocytogenes* CECT911 and *Listeria ivanovii* CECT913) [110]. For this purpose, bacteria were stabbed into MRS agar (1.5%, *w*/*v*) using sterile sticks, and after incubation at 30 °C for 5 h, the corresponding semisolid medium (0.8% agar, *w*/*v*) inoculated with the indicator microorganism (1 × 10^6^ cfu/mL) was poured over the plates. After incubation at 30 °C for 16–24 h, the inhibition halos formed around the isolates under study were measured (mm) [114]. A total of 66 isolates were pre-selected based on their antimicrobial activity.

### 4.3. Taxonomic Identification of Bacterial Isolates

The identification of the isolates was achieved through comprehensive sequencing of the *16S rDNA*. To accomplish this, total DNA was extracted from the isolates that had been pre-selected based on their antimicrobial properties, utilising the InstaGene Matrix (Bio-Rad Laboratories, Inc., Hercules, CA, USA) according to the manufacturer’s guidelines. The amplification of the 16S *rDNA* gene was performed using PCR, incorporating 25 µL of DreamTaq Hot Start PCR Master Mix 2× (Thermo Scientific, Waltham, MA, USA), 0.5 µM of the forward primer fD1 (5′-AGAGAGAGTTTGGATCCTGGCTCAG-3′), 0.5 µM of the reverse primer rD1 (5′-TAAGGAGGAGGAGGAGGTGATCCAGCC-3′), 50–100 ng of purified DNA and 19 µL of molecular biology-grade water (Thermo Scientific, Waltham, MA, USA) [115]. The PCR mixtures underwent a series of amplification cycles, beginning with an initial denaturation at 95 °C for 3 min, followed by 35 cycles consisting of denaturation at 95 °C for 30 s, hybridisation at 60 °C for 30 s, elongation at 72 °C for 1 min and concluding with a final elongation at 72 °C for 5 min using a thermal cycler (Eppendorf, Hamburg, Germany). The resulting amplicons were then purified with the NucleoSpin^®^ Gel and PCR Clean-up kit (Macherey-Nagel™, Düren, Germany) and forwarded to Eurofins Genomics (Ebersberg, Germany) for DNA sequencing. The nucleotide sequences obtained were analysed using the NCBI BLAST nucleotide server (https://blast.ncbi.nlm.nih.gov/, accessed on 15 April 2024) for taxonomic identification. Out of the 66 identified isolates, only LAB (*n* = 35) were selected for further characterization.

### 4.4. Genetic Diversity Analysis by Enterobacterial Repetitive Intergenic Consensus-PCR (ERIC-PCR)

To examine the genetic diversity among the various LAB isolated from European hake, ERIC-PCR was conducted using the primers ERIC-1R (5′-ATGTAAGCTCCTGGGGGGGGGGGGATTCAC-3′) and ERIC-2 (5′-AAGTAAGTG ACTGGGGGGGGGTGAGCG-3′), as previously detailed by Araújo et al. (2015) [15]. PCR reactions of 50 µL were prepared with 25 µL of MyTaq Mix (Bioline Reagents, Ltd., London, UK), 0.7 µM of each primer, 50–100 ng of purified DNA, 3 µM of MgCl2 and 19 µL of molecular biology-grade water. The PCR cycles included an initial denaturation step at 95 °C for 1 min, followed by 35 cycles consisting of denaturation, annealing and elongation at 95 °C for 15 s, 46 °C for 15 s and 72 °C for 10 s and concluding with a final elongation step at 72 °C for 4 min in a thermal cycler. The amplified products were subjected to electrophoresis at 90 V for 60 min using an electrophoresis chamber (Bio-Rad Laboratories, Inc., Hercules, CA, USA), and band visualization was performed using a ChemiDoc Imaging System (Bio-Rad Laboratories, Inc.) with HyperLadder 100 bp (Bioline Reagents, Ltd., London, UK) as a molecular weight marker. For the analysis of ERIC types, clustering and dendrogram construction, Phoretix v.5.0 software (Nonlinear Dynamics Ltd., Newcastle upon Tyne, UK) was utilised.

### 4.5. Biofilm Formation and Quantification

In order to evaluate the biofilm formation, a microtiter assay, previously described by Oniciuc E.-A. et al. (2016) [116], was performed. Each isolate was grown on MRS agar plates (Oxoid) and incubated at 30 °C for 24 h. Then, a pair of colonies were transferred to 3 mL tubes of TSB (Tryptic Soy Broth, Oxoid) and incubated at 30 °C for 16 ± 1 h with continuous shaking at 120 rpm (ES- 80 Shaker-incubator, Grant Instruments). Then, 200 µL of bacterial suspension with a concentration of 1 × 10^6^ cfu/mL was added to each well of the 96-well flat-bottom microplate (Thermo Scientific). In all plates, *Staphylococcus aureus* CECT794 was included as a positive control and TSB without bacterial inoculum as a negative control. The plates were incubated in aerobiosis for 24 h at 30 °C. All experiments had sixteen replicates.

For the quantification of biofilm production, the crystal violet (CV) staining method was used, as previously described by Peeters et al. (2008) [117], with some modifications. Once the incubation was finished, the unattached bacterial cells were removed by eliminating the medium from each well and washing the plates twice with sterile distilled water. The plates were left to dry at room temperature for 30 min. To fix the biofilms, 100 µL of methanol (VWR International) was added to each well. After 15 min, the methanol was discarded, the plates were dried at room temperature for 10 min and 100 µL of 1% (*v*/*v*) CV was added to each well. After 10 min, the CV was removed, and the plates were washed twice with distilled water to eliminate excess dye and then dried. In order to solubilize the CV, 100 µL of 33% (*v*/*v*) acetic acid was subsequently added and the absorbance was measured at 570 nm using a BioTek ELx808U microplate reader (BioTek, Winooski, VT, USA). To standardise the results, the biofilm formation capacity of each isolate was normalised, assuming that the positive control *S. aureus* CECT794 produces 100% biofilm. GraphPad Prism 8 (GraphPad Software, San Diego, CA, USA) was used for data processing, analysis and graphical representation. The normal distribution of all the data was verified using the Shapiro–Wilk test. Statistical analyses were then performed using paired Student’s *t*-tests.

### 4.6. Antibiotic Susceptibility Testing

Antibiotic susceptibility was determined by a broth microdilution test in order to determine the minimum inhibitory concentration (MIC) of 14 antibiotics: ampicillin (0.25–16 µg/mL); vancomycin (1–64 µg/mL); gentamicin (0.5–32 µg/mL); kanamycin (1–128 µg/mL); streptomycin (1–64 µg/mL); erythromycin (0.25–16 µg/mL); clindamycin (0.25–16 µg/mL); tetracycline (0.5–32 µg/mL); chloramphenicol (1–64 µg/mL); florfenicol (0.125–8 µg/mL); oxytetracycline (0.03–4 µg/mL); flumequine (0.125–8 µg/mL); amoxicillin (0.125–8 µg/mL); trimethoprim-sulfamethoxazole (0.0625/1.1875–4/76 µg/mL). These antibiotics were chosen according to the EFSA Technical Panel on Additives and Products or Substances used in Animal Feed (FEEDAP) guidelines on Guidance on the characterisation of microorganisms used as feed additives or production organisms [118]. In addition, antibiotics frequently used in aquaculture were also evaluated [119,120,121].

Antimicrobial susceptibility testing was performed in accordance with the European Committee on Antimicrobial Susceptibility Testing (EUCAST) [122,123] and Clinical and Laboratory Standards Institute (CLSI) [44,45,120]. Individual colonies of each strain were chosen from a culture grown in MRS agar at 30 °C for 16 h and incorporated into 10 mL of saline solution (0.9% NaCl; *w*/*v*) until its optical density was adjusted to a value of 0.5 according to the McFarland scale (ca. 1.5 × 10^8^ cfu/mL). Then, they were diluted (1:100) in Mueller–Hinton medium to obtain ca. 1.5 × 10^6^ cfu/mL. Serial two-fold dilutions of each antibiotic were prepared in 96-well microtiter plates (Thermo Scientific), and 50 μL of the bacterial culture was added to each well, obtaining a final concentration of ca. 7.5 × 10^5^ cfu/mL. Subsequently, the plates were shaken for 3 min and, finally, incubated at 30 °C for 24 h [44,45,124]. After the incubation, the MIC was determined for each antibiotic, which is defined as the minimum concentration that inhibits bacterial growth [43]. MICs were compared to cut-off values established by [43] and/or the CLSI breakpoints [44,45,124]. Isolates were considered resistant when the MIC for one specific antibiotic was higher than the most restrictive cut-off value. In relation to this, when an exact cut-off value for some bacterial species evaluated in this study had not previously been described, it was adjusted with the cut-off value corresponding to a related species. Specifically, the EFSA cut-off values for *Lc. garvieae* were compared with those for *Lactococcus lactis*, *Streptococcus salivarius* with *Streptococcus thermophilus*, *Enterococcus avium* with *Enterococcus faecium* and *Lactobacillus sakei* with *Lactobacillus* facultative heterofermentative. Quality control was performed with the strains *S. aureus* CECT794 and *Enterococcus faecalis* CECT795. Two independent experiments were performed in duplicate.

### 4.7. Evaluation of Haemolytic and Gelatinase Activities

Haemolytic activity was determined using the method previously described by Eaton and Gasson (2001) [125]. The isolates were inoculated in MRS broth and incubated at 30 °C in aerobiosis for 16 h. Subsequently, bacteria were streaked onto Columbia agar plates supplemented with horse blood (5%, *v/v*) (BioMérieux, Marcy L’Etoile, France). After incubation at 30 °C for 24 h, the α- and β-haemolysis were revealed by the appearance of greenish halos and clear zones, respectively, around the colonies. *E. faecalis* SDP10 and *E. faecalis* P4 strains were used as positive controls. Two independent experiments were performed.

Gelatinase production was determined using the method previously described by Eaton and Gasson (2001) [125]. The isolates were inoculated in MRS broth and incubated in aerobiosis at 30 °C for 16 h. Subsequently, bacteria were seeded onto Todd–Hewitt (Oxoid) agar plates (1.5%, *w/v*) containing 3% (*w/v*) of gelatine (Oxoid) and incubated at 30 °C for 24 h. After incubation, the plates were kept at 4 °C for 5 h. The presence of a zone of turbidity (gelatin hydrolysis) around the inoculation streak was considered indicative of proteolytic activity. *E. faecalis* SDP10 and *E. faecalis* P4 strains were used as positive controls. Two independent experiments were performed.

## 5. Conclusions

The intestine and faeces of Northeast Atlantic hake are suitable ecological niches for the isolation of LAB (*Lactobacillus* spp., *Lactococcus* spp., *Leuconostoc* spp. and *Streptococcus* spp.) with antimicrobial activity against ichthyopathogens. After the different analyses carried out in this work, only five strains (11.4%), *St. salivarius* MDI13, *St. salivarius* MDI20, *E. avium* MEI4, *E. avium* MGH6 and *Lb. sakei* MEI5, have been selected for future studies as putative safe probiotic candidates for aquaculture, since all of them were susceptible to antibiotics and none showed any of the tested virulence factors (haemolytic and proteolytic activities). This approach could contribute to the development of sustainable aquaculture in the context of One Health, not only for the prevention and control of the most relevant ichthyopathologies but also to contribute to the health, immune status, productivity and economic profitability of aquaculture farms. However, additional genetic analyse*s*, including the detection of antibiotic resistance genes, plasmids, mobile genetic elements and virulence factors, along with in vivo assays, are necessary to further validate our findings and confirm the suitability and safety of these strains as probiotics.

## Figures and Tables

**Table 1:** Susceptibility of the 35 LAB isolates to the evaluated antibiotics.

Antibiotics	Species (Number of Tested Isolates)	Number of Strains with the Indicated MIC (µg/mL) ^a^												EFSA/CLSI Cut-Off Values (µg/mL)
		0.062	0.125	0.25	0.5	1	2	4	8	16	32	64	128	
Ampicillin	*Lc. garvieae* (21)						6	**15**						**>2/≥4**
	*St. salivarius* (6)						2	**1**		**3**				**>2/≥8**
	*E. avium* (6)						2	**2**	**2**					**>2/≥16**
	*Lb. sakei* (1)							1						**>4/Na**
	*Lt. carnosum* (1)							**1**						**>2/Na**
Vancomycin ^b^	*Lc. garvieae* (21)					21								**>4/≥32**
	*St. salivarius* (6)					6								**>4/Na**
	*E. avium* (6)					4	2							**>4/≥32**
Gentamicin	*Lc. garvieae* (21)				1		1	5	11	**3**				**>32/≥16**
	*St. salivarius* (6)							1	4	**1**				**>32/≥16**
	*E. avium* (6)				3	3								**>32/≥16**
	*Lb. sakei* (1)						1							**>16/≥16**
	*Lt. carnosum* (1)					1								**>16/≥16**
Kanamycin	*Lc. garvieae* (21)						1				1	**15**	**4**	**>64/≥64**
	*St. salivarius* (6)									1	2	**3**		**Nr/≥64**
	*E. avium* (6)					2		3	1					**>1.024/≥64**
	*Lb. sakei* (1)								1					**>64/≥64**
	*Lt. carnosum* (1)							1						**>16/≥64**
Streptomycin	*Lc. garvieae* (21)						1				3	**17**		**>32/Na**
	*St. salivarius* (6)							1			5			**>64/Na**
	*E. avium* (6)							5		1				**>128/Na**
	*Lb. sakei* (1)						1							**>64/Na**
	*Lt. carnosum* (1)											1		**>64/Na**
Erythromycin	*Lc. garvieae* (21)			6	15									**>1/≥8**
	*St. salivarius* (6)			2	2					**2**				**>2/≥1**
	*E. avium* (6)			2	4									**>4/≥8**
	*Lb. sakei* (1)			1										**>1/≥8**
	*Lt. carnosum* (1)				1									**>1/≥8**
Clindamycin	*Lc. garvieae* (21)									**21**				**>1/≥4**
	*St. salivarius* (6)			6										**>2/≥4**
	*E. avium* (6)					1	4			**1**				**>4/≥4**
	*Lb. sakei* (1)			1										**>4/≥2**
	*Lt. carnosum* (1)					1								**>1/≥4**
Tetracycline	*Lc. garvieae* (21)				2	9	9	1						**>4/≥8**
	*St. salivarius* (6)				4					**2**				**>4/≥8**
	*E. avium* (6)				6									**>4/≥16**
	*Lb. sakei* (1)						1							**>8//≥16**
	*Lt. carnosum* (1)					1								**>8/≥16**
Chloramphenicol	*Lc. garvieae* (21)					1	1	17	2					**>8/≥32**
	*St. salivarius* (6)					4	2							**>8/≥16**
	*E. avium* (6)					4	2							**>16/≥32**
	*Lb. sakei* (1)							1						**>4/≥32**
	*Lt. carnosum* (1)						1							**>4/≥32**
Florfenicol	*Lc. garvieae* (21)							10	**11**					**Na/≥8**
	*St. salivarius* (6)					4	2							**Na/≥8**
	*E. avium* (6)				3	1	1	1						**Na/≥8**
	*Lb. sakei* (1)						1							**Na/≥8**
	*Lt. carnosum* (1)						1							**Na/≥8**
Oxytetracycline	*Lc. garvieae* (21)		1	8	4	1	1	7						**Na/≥16**
	*St. salivarius* (6)		5					1						**Na/≥8**
	*E. avium* (6)			4	2									**Na/≥16**
	*Lb. sakei* (1)						1							**Na/≥16**
	*Lc. carnosum* (1)	1												**Na/≥16**
Flumequine	*Lc. garvieae* (21)						2	4	16					**Na**
	*St. salivarius* (6)								6					**Na**
	*E. avium* (6)						1	3	2					**Na**
	*Lb. sakei* (1)								1					**Na**
	*Lt. carnosum* (1)		1											**Na**
Amoxicillin	*Lc. garvieae* (21)			3	8	10								**Na/≥8**
	*St. salivarius* (6)				5	1								**Na/≥16**
	*E. avium* (6)			2	1	3								**Na**
	*Lb. sakei* (1)					1								**Na**
	*Lt. carnosum* (1)		1											**Na**
**Antibiotics**	**Species (Number of Tested Isolates)**	**Number of Strains with the Indicated MIC (µg/mL) ^a^**												**EFSA/CLSI** **Cut-off Values (µg/mL)**
		**0.0625/1.1875**		**0.125/2.375**		**0.25/4.75**		**0.5/9.5**		**1/19**		**2/38**	**4/76**	
Trimethoprim- sulfamethoxazole	*Lc. garvieae* (21)												**21**	**Na/≥4–76**
	*St. salivarius* (6)							5		1				**Na/≥4–76**
	*E. avium* (6)	6												**Na/≥4–76**
	*Lb. sakei* (1)											1		**Na/≥4–76**
	*Lt. carnosum* (1)												**1**	**Na/≥4–76**

^a^ Shaded areas show the range of dilutions tested for each antibiotic. MICs higher than the cut-off values established by the EFSA [38] and/or equal to or higher than the CLSI breakpoints [44,45] are underlined and bolded. ^b^ According to the EFSA [43], the cut-off value for vancomycin is not required for *Lactobacillus* facultative heterofermentative, nor *Leuconostoc* spp. Na: not available. Nr: not required for *Streptococcus thermophilus*.

## Data Availability

The original contributions presented in this study are included in the article/Supplementary Materials. Further inquiries can be directed to the corresponding authors.

## Supplementary Materials

The following supporting information can be downloaded at https://www.mdpi.com/article/10.3390/antibiotics14050469/s1: Table S1. Direct antimicrobial activity of the 66 isolates from European hakes against pathogenic bacteria by a Stab-On-Agar Test (SOAT).

## References

[1] Food and Agriculture Organization (FAO) (2020). The State of WORLD Fisheries and Aquaculture 2020.

[2] Hunter M.C., Smith R.G., Schipanski M.E., Atwood L.W., Mortensen D.A. (2017). Agriculture in 2050: Recalibrating targets for sustainable intensification. BioScience.

[3] Food and Agriculture Organization (FAO) (2022). The State of World Fisheries and Aquaculture 2022. https://www.fao.org/documents/card/en/c/cc0461es.

[4] Álvarez-Pellitero P. (2008). Fish immunity and parasite infections: From innate immunity to immunity to immunoprophylactic prospects. Vet. Immunol. Immunopathol..

[5] Almeida A., Cunha A., Gomes N.C., Alves E., Costa L., Faustino M.A. (2009). Phage therapy and photodynamic therapy: Low environmental impact approaches to inactivate microorganisms in fish farming plants. Mar. Drugs.

[6] Zhang Q., Gui J.F. (2015). Virus genomes and virus-host interactions in aquaculture animals. Sci. China Life Sci..

[7] Kibenge F.S. (2019). Emerging viruses in aquaculture. Curr. Opin. Virol..

[8] Abowei F.N., Briyai O.F., Bassey S.E. (2011). A review of some basic parasite diseases in culture fisheries: Flagellids, dinoflagellides and ichthyophthriasis, ichtyobodiasis, coccidiosis trichodiniasis, heminthiasis, hirudinea infestation, crustacean parsite and ciliates. Br. J. Pharmacol. Toxicol..

[9] Stentiford G.D., Bateman I.J., Hinchliffe S.J., Bass D., Hartnell R., Santos E.M., Devlin M.J., Feist S.W., Taylor N.G.H., Verner-Jeffreys D.W. (2020). Sustainable aquaculture through the One Health lens. Nat. Food.

[10] Valle A., Leiro J.M., Pereiro P., Figueras A., Novoa B., Dirks R.P.H., Lamas J. (2020). Interactions between the parasite *Philasterides dicentrarchi* and the immune system of the turbot *Scophthalmus maximus*. A transcriptomic analysis. Biology.

[11] Moreira M., Schrama D., Farinha A.P., Cerqueira M., Raposo de Magalhães C., Carrilho R., Rodrigues P. (2021). Fish pathology research and diagnosis in aquaculture of farmed fish; a proteomics perspective. Animals.

[12] Food and Agriculture Organization (FAO) (2024). The State of World Fisheries and Aquaculture 2024. Blue Transformation in Action. https://openknowledge.fao.org/items/06690fd0-d133-424c-9673-1849e414543d.

[13] Chen J., Sun R., Pan C., Sun Y., Mai B., Li Q.X. (2020). Antibiotics and Food Safety in Aquaculture. J Agric Food Chem..

[14] Muñoz-Atienza E., Gómez-Sala B., Araújo C., Campanero C., del Campo R., Hernández P.E., Herranz C., Cintas L.M. (2013). Antimicrobial activity, antibiotic susceptibility and virulence factors of Lactic Acid Bacteria of aquatic origin intended for use as probiotics in aquaculture. BMC Microbiol..

[15] Araújo C., Muñoz-Atienza E., Ramírez M., Poeta P., Igrejas G., Hernández P.E., Herranz C., Cintas L.M. (2015). Safety assessment, genetic relatedness and bacteriocin activity of potential probiotic *Lactococcus lactis* strains from rainbow trout (*Oncorhynchus mykiss*, Walbaum) and rearing environment. Eur. Food Res. Technol..

[16] World Health Organization (WHO) (2020). Resistencia a Los Antimicrobianos. https://www.who.int/es/news-room/fact-sheets/detail/antimicrobial-resistance.

[17] El-Saadony M.T., Alagawany M., Patra A.K., Kar I., Tiwari R., Dawood M.A.O., Dhama K., Abdel-Latif H.M.R. (2021). The functionality of probiotics in aquaculture: An overview. Fish Shellfish Immunol..

[18] Nayak S.K. (2020). Current prospects and challenges in fish vaccine development in India with special reference to Aeromonas hydrophila vaccine. Fish Shellfish Immunol..

[19] Kumar A., Middha S.K., Menon S.V., Paital B., Gokarn S., Nelli M., Rajanikanth R.B., Chandra H.M., Mugunthan S.P., Kantwa S.M. (2024). Current challenges of vaccination in fish health management. Animals.

[20] Toranzo A., Magariños B., Romalde J.L., Barja J.L. (2009). Present and future of aquaculture vaccines against fish bacterias diseases. Options Méditerranéennes.

[21] Adams A. (2019). Progress, challenges and opportunities in fish vaccine development. Fish Shellfish Immunol..

[22] Collins C., Lorenzen N., Collet B. (2019). DNA vaccination for finfish aquaculture. Fish Shellfish Immunol..

[23] Pérez-Sánchez T., Ruiz-Zarzuela I., de Blas I., Balcázar J.L. (2014). Probiotics in aquaculture: A current assessment. Rev. Aquac..

[24] Gómez-Sala B., Feito J., Hernández P.E., Cintas L.M., Vinderola G., Ouwehand A.C., Salminen S., von Wright A. (2019). Lactic Acid Bacteria in aquatic Environments and Their Applications.

[25] Thatcher C., Høj L., Bourne D.G. (2022). Probiotics for coral aquaculture: Challenges and considerations. Curr. Opin. Biotechnol..

[26] EFSA (2021). Statement on the requirements for whole genome sequence analysis of microorganisms intentionally used in the food chain. EFSA J..

[27] Commission Implementing Regulation (EU) (2020). 2020/151 of 4 February 2020 concerning the authorisation of *Pediococcus acidilactici* CNCM I-4622 as a feed additive for all porcine species for fattening and for breeding other than sows, all avian species, all fish species and all crustaceans and repealing Regulations (EC) No 911/2009, (EU) No 1120/2010 and (EU) No 212/2011 and Implementing Regulations (EU) No 95/2013, (EU) No 413/2013 and (EU) 2017/2299 (holder of authorisation Danstar Ferment AG represented in the Union by Lallemand SAS). Off. J. Eur. Union.

[28] Todorov S.D., Lima J.M.S., Bucheli J.E.V., Popov I.V., Tiwari S.K., Chikindas M.L. (2024). Probiotics for Aquaculture: Hope, Truth, and Reality. Probiot. Antimicrob. Proteins.

[29] Gillor O., Etzion A., Riley M.A. (2008). The dual role of bacteriocins as anti- and probiotics. Appl. Microbiol. Biotechnol..

[30] Ringø E., Hoseinifar S.H., Ghosh K., Doan H.V., Beck B.R., Song S.K. (2018). Lactic acid bacteria in finfish-an update. Front. Microbiol..

[31] Ringø E., Van Doan H., Lee S.H., Soltani M., Hoseinifar S.H., Harikrishnan R., Song S.K. (2020). Probiotics, lactic acid bacteria and bacilli: Interesting supplementation for aquaculture. J. App. Microbiol..

[32] Zorriehzahra M.J., Delshad S.T., Adel M., Tiwari R., Karthik K., Dhama K., Lazado C.C. (2016). Probiotics as beneficial microbes in aquaculture: An update on their multiple modes of action: A review. Vet Q..

[33] Vine N.G., Leukes W.D., Kaiser H. (2006). Probiotics in marine larviculture. FEMS Microbiol. Rev..

[34] Ringø E., Løvmo L., Kristiansen M., Bakken Y., Salinas I., Myklebust R., Olsen R.E., Mayhew T.M. (2010). Lactic acid bacteria *vs.* pathogens in the gastrointestinal tract of fish: A review. Aquac. Res..

[35] Dobson A., Cotter P.D., Ross R.P., Hill C. (2012). Bacteriocin production: A probiotic trait?. Appl. Environ. Microbiol..

[36] Ołdak A., Zielińska D. (2017). Bacteriocins from lactic acid bacteria as an alternative to antibiotics. Postep. Hig. Med. Dosw..

[37] Hernández-González J.C., Martínez-Tapia G.A., Lazcano-Hernández B.E., García-Pérez Castrejón-Jiménez N.S. (2021). Bacteriocins from lactic acid bacteria. A powerful alternative as antimicrobials, probiotics, and immunomodulators in veterinary medicine. Animals.

[38] Uniacke-Lowe S., Collins F.W.J., Hill C., Ross R.P. (2023). Bioactivity screening and genomic analysis reveals deep-sea fish microbiome isolates as sources of novel antimicrobials. Mar. Drugs..

[39] Ministerio de Agricultura, Pesca y Alimentación (MAPA) (2022). Estadísticas Pesqueras: Pesca Marítima. https://www.mapa.gob.es/es/estadistica/temas/estadisticas-pesqueras/pesca-maritima/.

[40] Ringø E., Jutfelt F., Kanapathippillai P., Bakken Y., Sundell K., Glette J., Mayhew T.M., Myklebust R., Olsen R.E. (2004). Damaging effect of the fish pathogen *Aeromonas salmonicida* ssp. salmonicida on intestinal enterocytes of Atlantic salmon (Salmo salar, L.). Cell. Tissue. Res..

[41] Mabrok M., Algammal A.M., Sivaramasamy E., Hetta H.F., Atwah B., Alghamdi S., Fawzy A., Avendaño-Herrera R., Rodkhum C. (2023). Tenacibaculosis caused by *Tenacibaculum maritimum*: Updated knowledge of this marine bacterial fish pathogen. Front. Cell. Infect. Microbiol..

[42] Nho S.W., Hikima J., Cha I.S., Park S.B., Jang H.B., del Castillo C.S., Kondo H., Hirono I., Aoki T., Jung T.S. (2011). Complete genome sequence and immunoproteomic analyses of the bacterial fish pathogen *Streptococcus parauberis*. J. Bacteriol..

[43] FEEDAP (2018). Guidance on the characterization of microorganisms used as feed additives or as production organisms. EFSA J..

[44] Clinical and Laboratory Standards Institute (CLSI) (2015). M45 Methods for Antimicrobial Dilution and Disk Susceptibility Testing of Infrequently Isolated or Fastidious Bacteria.

[45] Clinical and Laboratory Standards Institute (CLSI) (2020). M100 Performance Standards for Antimicrobial Susceptibility Testing.

[46] Gónzalez C., Encinas J.P., García-López L.M., Otero A. (2020). Characterisation and identification of lactic acid bacteria from freshwater fishes. Food Microbiol..

[47] Ringø E., Holzapfel W. (2000). Identification and characterization of carnobacteria associated with the gills of Atlantic salmon (*Salmo salar* L.). Syst. Appl. Microbiol..

[48] Elidrissi A., Ezzaky Y., Boussif K., Achemchem F. (2023). Isolation and characterization of bioprotective lactic acid bacteria from Moroccan fish and seafood. Braz. J. Microbiol..

[49] Cheriet S., Lengliz S., Romdhani A., Hynds P., Abbassi M.S., Ghrairi T. (2023). Selection and characterization of bacteriocinogenic Lactic Acid Bacteria from the intestine of gilthead seabream (*Sparus aurata*) and whiting fish (*Merlangius merlangus*): Promising strains for aquaculture probiotic and food bio-preservation. Life.

[50] Seppola M., Olsen R.E., Sandeker E., Kanapathippillai P., Holzapfel W., Ringø E. (2006). Random amplification of polymorphic DNA (RAPD) typing of carnobacteria isolated from hindgur chamber and large intestine of atlantic cod (*Gadus morhua* L.). Syst. Appl. Microbiol..

[51] Pinto A., Fernandes L., Pinto C., Albano H., Castillo F., Teixeira P., Gibbs A. (2009). Characterization of anti-*Listeria* bacteriocins isolated from shellfish: Potencial antimicrobials to control non-fermented seafood. Int. J. Food. Microbiol..

[52] Lauzon H.L., Ringo E. (2012). Prevalence and application of lactic acid bacteria in aquatic environments. Lactic Acid Bacteria: Microbiological and Functional Aspects.

[53] Gómez-Sala B., Herranz C., Díaz-Freitas B., Hernández P.E., Sala A., Cintas L.M. (2016). Strategies to increase the hygienic and economic value of fresh fish: Biopreservation using lactic acid bacteria of marine origin. Int. J. Food Microbiol..

[54] Sahoo T.K., Jena P.K., Nagar N., Patel A.K., Seshadri S. (2015). In vitro evaluation of probiotic properties of lactic acid bacteria from the gut of *Labeo rohita* and *Catla catla*. Probiotics Antimicrob. Proteins.

[55] Patel P., Patel B., Amaresan N., Joshi B., Shah R., Krishnamurthy R. (2020). Isolation and characterization of *Lactococcus garvieae* from the fish gut for *in vitro* fermentation with carbohydrates from agro-industrial waste. Biotechnol. Rep..

[56] Araújo C., Muñoz-Atienza E., Nahuelquín Y., Poeta P., Igrejas G., Hernández P.E., Herranz C., Cintas L.M. (2015). Inhibition of fish pathogens by the microbiota from rainbow trout (*Oncorhynchus mykiss*, Walbaum) and rearing environment. Anaerobe.

[57] Phupaboon S., Hashim F.J., Phumkhachorn P., Rattanachaikunsopon P. (2023). Molecular and biotechnological characteristics of proteolytic activity from *Streptococcus thermophiles* as a proteolytic lactic acid bacteria to enhance protein-derived bioactive peptides. AIMS Microbiol..

[58] Hoseinifar S.H., Sun Y.Z., Wang A., Zhou Z. (2018). Probiotics as means of diseases control in aquaculture, a review of current knowledge and future perspectives. Front. Microbiol..

[59] Ringø E., Li X., Doan H., Ghosh K. (2022). Interesting probiotic bacteria other than the more widely used lactic acid bacteria and bacilliin finfish. Front. Mar. Sci..

[60] Simón R., Docando F., Nu.ez-Ortiz N., Tafalla C., Díaz-Rosales P. (2021). Mechanisms used by probiotics to confer pathogen resistance to teleost fish. Front. Immunol..

[61] Sumon M.A.A., Molla M.H.R., Hakeem I.J., Ahammad F., Amran R.H., Jamal M.T., Gabr M.H., Islam M.S., Alam M.T., Brown C.L. (2022). Epigenetics and probiotics application toward the modulation of fish reproductive performance. Fishes.

[62] Cintas L.M., Casaus M.P., Herranz C., Nes I.F., Hernández P.E. (2001). Review: Bacteriocins of lactic acid bacteria. Food Sci. Technol. Int..

[63] Cotter P.D., Ross R.P., Hill C. (2013). Bacteriocins—A viable alternative to antibiotics?. Nat. Rev. Microbiol..

[64] Zhang Y., Xu D., Shi L., Cai R., Li C., Yan H. (2018). Association between *agr* type, virulence factors, biofilm formation and antibiotic resistance of *Staphylococcus aureus* isolates from pork production. Front. Microbiol..

[65] Rodríguez-López P., Filipello V., Di Ciccio P.A., Pitozzi A., Ghidini S., Scali F., Ianieri A., Zanardi E., Losio M.N., Simon A.C. (2020). Assessment of the antibiotic resistance profile, genetic heterogeneity and biofilm production of Methicillin-Resistant *Staphylococcus aureus* (MRSA) isolated from the Italian swine production chain. Foods.

[66] Cho J.A., Roh Y.J., Son H.R., Choi H., Lee J.W., Kim S.J., Lee C.H. (2022). Assessment of the biofilm-forming ability on solid surfaces of periprosthetic infection-associated pathogens. Sci. Rep..

[67] Contente D., Díaz-Formoso L., Feito J., Gómez-Sala B., Costas D., Hernández P.E., Muñoz-Atienza E., Borrero J., Poeta P., Cintas L.M. (2024). Antimicrobial activity, genetic relatedness, and safety assessment of potential probiotic lactic acid bacteria isolated from a rearing tank of rotifers (*Brachionus plicatilis*) used as live feed in fish larviculture. Animals.

[68] Rezaei Z., Khanzadi S., Salari A. (2021). Biofilm formation and antagonistic activity of *Lacticaseibacillus rhamnosus* (PTCC1712) and *Lactiplantibacillus plantarum* (PTCC1745). AMB Express.

[69] Mirzabekyan S., Harutyunyan N., Manvelyan A., Malkhasyan L., Balayan M., Miralimova S., Chikindas M.L., Chistyakov V., Pepoyan A. (2023). Fish probiotics: Cell surface properties of fish intestinal lactobacilli and *Escherichia coli*. Microorganisms.

[70] Sharma S., Mohler J., Mahajan S.D., Schwartz S.A., Bruggemann L., Aalinkeel R. (2023). Microbial Biofilm: A review on formation, infection, antibiotic resistance, control measures, and innovative treatment. Microorganisms.

[71] Chamignon C., Guéneau V., Medina S., Deschamps J., Gil-Izquierdo A., Briandet R., Mousset P.-Y., Langella P., Lafay S., Bermúdez-Humarán L.G. (2020). Evaluation of the probiotic properties and the capacity to form biofilms of various *Lactobacillus* strains. Microorganisms.

[72] Shaaban M., Abd El-Rahman O.A., Al-Qaidi B., Ashour H.M. (2020). Antimicrobial and antibiofilm activities of probiotic Lactobacilli on antibiotic-resistant *Proteus mirabilis*. Microorganisms.

[73] Feito J., Araújo C., Arbulu S., Contente D., Gómez-Sala B., Díaz-Formoso L., Muñoz-Atienza E., Borrero J., Cintas L.M., Hernández P.E. (2023). Design of *Lactococcus lactis* strains producing garvicin A and/or garvicin Q, either alone or together with nisin A or nisin Z and high antimicrobial activity against *Lactococcus garvieae*. Foods.

[74] Elliott J.A., Facklam R.R. (1996). Antimicrobial susceptibilities of *Lactococcus lactis* and *Lactococcus garvieae* and a proposed method to discriminate between them. J. Clin. Microbiol..

[75] Walther C., Rossano A., Thomann A., Perreten V. (2008). Antibiotic resistance in *Lactococcus* species from bovine milk: Presence of a mutated multidrug transporter *mdt(A*) gene in susceptible *Lactococcus garvieae* strains. Vet. Microbiol..

[76] Plumed-Ferrer C., Barberio A., Franklin-Guild R., Werner B., McDonough P., Bennett J., Gioia G., Rota N., Welcome F., Nydam D.V. (2015). Antimicrobial susceptibilities and random amplified polymorphic DNA-PCR fingerprint characterization of *Lactococcus lactis* ssp. *lactis* and *Lactococcus garvieae* isolated from bovine intramammary infections. J. Dairy Sci..

[77] Fortina M.G., Ricci G., Foschino R., Picozzi C., Dolci P., Zeppa G., Cocolin L., Manachini P.L. (2007). Phenotypic typing, technological properties and safety aspects of *Lactococcus garvieae* strains from dairy environments. J. Appl. Microbiol..

[78] Raissy M., Moumeni M. (2016). Detection of antibiotic resistance genes in some *Lactococcus garvieae* strains isolated from infected rainbow trout. Iran. J. Fish. Sci..

[79] de Oliveira R.P., Aragão B.B., de Melo R.P.B., da Silva D.M.S., de Carvalho R.G., Juliano M.A., Farias M.P.O., de Lira N.S.C., Mota R.A. (2022). Bovine mastitis in northeastern Brazil: Occurrence of emergent bacteria and their phenotypic and genotypic profile of antimicrobial resistance. Comp. Immunol. Microbiol. Infect. Dis..

[80] Torres-Corral Y., Santos Y. (2022). Predicting antimicrobial resistance of *Lactococcus garvieae*: PCR detection of resistance genes *versus* MALDI-TOF protein profiling. Aquaculture.

[81] Shahi N., Mallik S.K. (2020). Emerging bacterial fish pathogen *Lactococcus garvieae* RTCLI04, isolated from rainbow trout (*Oncorhynchus mykiss*): Genomic features and comparative genomics. Microb. Pathog..

[82] Lin Y., Han J., Barkema H.W., Wang Y., Gao J., Kastelic J.P., Han B., Qin S., Deng Z. (2023). Comparative genomic analyses of *Lactococcus garvieae* isolated from bovine mastitis in China. Microbiol. Spectr..

[83] Chaffanel F., Charron-Bourgoin F., Libante V., Leblond-Bourget N., Payot S. (2015). Resistance genes and genetic elements associated with antibiotic resistance in clinical and commensal isolates of *Streptococcus salivarius*. Appl. Environ. Microbiol..

[84] Palma T.H., Harth-Chú E.N., Scott J., Stipp R.N., Boisvert H., Salomão M.F., Theobaldo J.D., Possobon R.F., Nascimento L.C., McCafferty J.W. (2016). Oral cavities of healthy infants harbour high proportions of *Streptococcus salivarius* strains with phenotypic and genotypic resistance to multiple classes of antibiotics. J. Med. Microbiol..

[85] Doumith M., Mushtaq S., Martin V., Chaudhry A., Adkin R., Coelho J., Chalker V., MacGowan A., Woodford N., Livermore D.M. (2017). Genomic sequences of *Streptococcus agalactiae* with high-level gentamicin resistance, collected in the BSAC bacteraemia surveillance. J. Antimicrob. Chemother..

[86] Barros R.R. (2021). Antimicrobial resistance among beta-hemolytic *Streptococcus* in Brazil: An Overview. Antibiotics.

[87] Wang J., Zhang Y.M., Lin M., Bao J., Wang G., Dong R., Zou P., Chen Y., Li N., Zhang T. (2023). Maternal colonization with group B *Streptococcus* and antibiotic resistance in China: Systematic review and meta-analyses. Ann. Clin. Microbiol. Antimicrob..

[88] Creti R., Imperi M., Khan U.B., Berardi A., Recchia S., Alfarone G., Gherardi G. (2024). Emergence of high-level gentamicin resistance in *Streptococcus agalactiae* hypervirulent serotype IV ST1010 (CC452) strains by acquisition of a novel integrative and conjugative element. Antibiotics.

[89] Alves-Barroco C., Rivas-García L., Fernandes A.R., Baptista P.V. (2020). Tackling multidrug resistance in streptococci—From novel biotherapeutic strategies to nanomedicines. Front. Microbiol..

[90] Du F., Lv X., Duan D., Wang L., Huang J. (2019). Characterization of a linezolid- and vancomycin-resistant *Streptococcus suis* isolate that harbors *optrA* and *vanG* operons. Front. Microbiol..

[91] Gergova R., Boyanov V., Muhtarova A., Alexandrova A. (2024). A review of the impact of streptococcal infections and antimicrobial resistance on human health. Antibiotics.

[92] Alzahrani O.M., Fayez M., Alswat A.S., Alkafafy M., Mahmoud S.F., Al-Marri T., Almuslem A., Ashfaq H., Yusuf S. (2022). Antimicrobial resistance, biofilm formation, and virulence genes in *Enterococcus* species from small backyard chicken flocks. Antibiotics.

[93] Fujii A., Kawada-Matsuo M., Nguyen-Tra Le M., Masuda K., Tadera K., Suzuki Y., Nishihama S., Hisatsune J., Sugawara Y., Kashiyama S. (2024). Antibiotic susceptibility and genome analysis of *Enterococcus* species isolated from inpatients in one hospital with no apparent outbreak of vancomycin-resistant *Enterococcus* in Japan. Microbiol. Immunol..

[94] Zhang X., Paganelli F.L., Bierschenk D., Kuipers A., Bonten M.J., Willems R.J., van Schaik W. (2012). Genome-wide identification of ampicillin resistance determinants in *Enterococcus faecium*. PLoS Genet..

[95] García-Solache M., Rice L.B. (2019). The *Enterococcus*: A model of adaptability to its environment. Clin. Microbiol. Rev..

[96] Gagetti P., Bonofiglio L., García Gabarrot G., Kaufman S., Mollerach M., Vigliarolo L., von Specht M., Toresani I., Lopardo H.A. (2019). Resistance to β-lactams in enterococci. Rev. Argent. Microbiol..

[97] Yang J., Chen Y., Dong Z., Zhang W., Liu L., Meng W., Li Q., Fu K., Zhou Z., Liu H. (2023). Distribution and association of antimicrobial resistance and virulence characteristics in *Enterococcus* spp. isolates from captive Asian elephants in China. Front. Microbiol..

[98] Noroozi N., Momtaz H., Tajbakhsh E. (2022). Molecular characterization and antimicrobial resistance of *Enterococcus faecalis* isolated from seafood samples. Vet. Med. Sci..

[99] Flórez A.B., Campedelli I., Delgado S., Alegría Á., Salvetti E., Felis G.E., Mayo B., Torriani S. (2016). Antibiotic susceptibility profiles of dairy leuconostoc, analysis of the genetic basis of atypical resistances and transfer of genes in vitro and in a food matrix. PLoS ONE.

[100] Salvetti E., Campedelli I., Larini I., Conedera G., Torriani S. (2021). Exploring Antibiotic resistance diversity in *Leuconostoc* spp. by a genome-based approach: Focus on the *lsa*A gene. Microorganisms.

[101] Raimondi S., Spampinato G., Candeliere F., Amaretti A., Brun P., Castagliuolo I., Rossi M. (2021). Phenotypic traits and immunomodulatory properties of *Leuconostoc carnosum* isolated from meat products. Front. Microbiol..

[102] Magiorakos A.P., Srinivasan A., Carey R.B., Carmeli Y., Falagas M.E., Giske C.G., Harbarth S., Hindler J.F., Kahlmeter G., Olsson-Liljequist B. (2012). Multidrug-resistant, extensively drug-resistant and pandrug-resistant bacteria: An international expert proposal for interim standard definitions for acquired resistance. Clin. Microbiol. Infect..

[103] Lehtinen S., Blanquart F., Lipsitch M., Fraser C. (2019). On the evolutionary ecology of multidrug resistance in bacteria. PLoS Pathog..

[104] Bernardeu M., Vernoux J.P., Henri-Dubernet S., Gueguen M. (2008). Safety assement of dairy organisms: The *Lactobacillus* genus. Int. J. Food Microbiol..

[105] Jaimee G., Halami P.M. (2016). Emerging resistance to aminoglycosides in lactic acid bacteria of food origin-an impending menace. Appl. Microbiol. Biotechnol..

[106] Reuben R.C., Roy P.C., Sarkar S.L., Alam R.U., Jahid I.K. (2019). Isolation, characterization, and assessment of lactic acid bacteria toward their selection as poultry probiotics. BMC Microbiol..

[107] Reuben R.C., Roy P.C., Sarkar S.L., Rubayet Ul Alam A.S.M., Jahid I.K. (2020). Characterization and evaluation of lactic acid bacteria from indigenous raw milk for potential probiotic properties. J. Dairy Sci..

[108] Okoye C.O., Nyaruaba R., Ita R.E., Okon S.U., Addey C.I., Ebido C.C., Opabunmi A.O., Okeke E.S., Chukwudozie K.I. (2022). Antibiotic resistance in the aquatic environment: Analytical techniques and interactive impact of emerging contaminants. Environ. Toxicol. Pharmacol..

[109] Akram F., Imtiaz M., Haq I.U. (2023). Emergent crisis of antibiotic resistance: A silent pandemic threat to 21st century. Microb. Pathog..

[110] Balcázar J.L., de Blas I., Ruiz-Zarzuela I., Vendrell D., Gironés O., Muzquiz J.L. (2007). Enhancement of the immune response and protection induced by probiotic lactic acid bacteria against furunculosis in rainbow trout (*Oncorhynchus mykiss*). FEMS Immunol. Med. Microbiol..

[111] Madsen K.T., Skov M.N., Gill S., Kemp M. (2017). Virulence factors associated with *Enterococcus faecalis* infective endocarditis: A mini review. Open Microbiol. J..

[112] Waters C.M., Antiporta M.H., Murray B.E., Dunny G.M. (2003). Role of the *Enterococcus faecalis* GelE protease in determination of cellular chain length, supernatant pheromone levels, and degradation of fibrin and misfolded surface proteins. J. Bacteriol..

[113] Unión Europea Denominaciones Comerciales. Mapa de las Zonas Pesqueras UE. Names of Sub-Areas and Divisions of FAO Fishing Areas 27 and 37. https://fish-commercial-names.ec.europa.eu/fish-names/fishing-areas_es.

[114] Cintas L.M., Rodríguez J.M., Fernández M.F., Sletten K., Nes I.F., Herández P.E., Holo H. (1995). Isolation and characterization of pediocin L50, a new bacteriocin from *Pediococcus acidilactici* with a broad inhibitory spectrum. Appl. Environ. Microbiol..

[115] Jaffrès E., Sohier D., Leroi F., Pilet M.F., Prévost H., Joffraud J.J., Dousset X. (2009). Study of the bacterial ecosystem in tropical cooked and peeled shrimps using a polyphasic approach. Int. J. Food Microbiol..

[116] Oniciuc E.-A., Cerca N., Nicolau A.I. (2016). Compositional Analysis of biofilms formed by *Staphylococcus aureus* isolated from food sources. Front. Microbiol..

[117] Peeters E., Nelis H.J., Coenye T. (2008). Comparison of multiple methods for quantification of microbial biofilms grown in microtiter plates. J. Microbiol. Methods.

[118] Rychen G., Aquilina G., Azimonti G., Bampidis V., Bastos M.L., Bories G., Chesson A., Cocconcelli P.S., Flachowsky G., EFSA FEEDAP Panel (EFSA Panel on Additives and Products or Substances used in Animal Feed) (2016). El Plan de Acción de la FAO Sobre la Resistencia a los Antimicrobianos 2016–2020. https://www.fao.org/3/i5996s/i5996s.pdf.

[119] Schar D., Klein E.Y., Laxminarayan R., Gilbert M., van Boeckel T.P. (2020). Global trends in antimicrobial use in aquaculture. Sci. Rep..

[120] Chowdhury S., Rheman S., Debnath N., Delamare-Deboutteville J., Akhtar Z., Ghosh S., Parveen S., Islam K., Islam M.A., Rashid M.M. (2022). Antibiotics usage practices in aquaculture in Bangladesh and their associate factors. One Health.

[121] Bondad-Reantaso M.G., MacKinnon B., Karunasagar I., Fridman S., Alday-Sanz V., Brun E., le Groumellec M., Li A., Surachetpong W., Karunasagar I. (2023). Reviews of alternatives to antibiotic use in aquaculture. Rev. Aquac..

[122] European Committee on Antimicrobial Susceptibility Testing (EUCAST) (2022). Expected Phenotypes Version 1.0. https://www.eucast.org/expert_rules_and_expected_phenotypes/expected_phenotypes.

[123] European Committee on Antimicrobial Susceptibility Testing (EUCAST) (2023). Breakpoint Tables for Interpretation of Mics and Zone Diameters; Version 13.1. https://www.eucast.org/fileadmin/src/media/PDFs/EUCAST_files/Breakpoint_tables/v_13.1_Breakpoint_Tables.pdf.

[124] Clinical and Laboratory Standards Institute (CLSI) (2002). M31-A2 Performance Standards for Antimicrobial Disk and Dilution Susceptibility Tests for Bacteria Isolated from Animals: Approved Standard.

[125] Eaton T.J., Gasson M.J. (2001). Molecular screening of *Enterococcus* virulence determinants and potential for genetic exchange between food and medical isolates. Appl. Environ. Microbiol..

