# Supplementary material for: Antimicrobial Activity, Genetic Diversity and Safety Assessment of Lactic Acid Bacteria Isolated from European Hakes (Merluccius merluccius, L.) Caught in the Northeast Atlantic Ocean
Source: Antibiotics (Basel). 2025 May 6;14(5):469. doi: 10.3390/antibiotics14050469 (PMC12108326; doi:10.3390/antibiotics14050469)
Supplement: Supplementary file 1 [file antibiotics-14-00469-s001.zip › antibiotics-3618574-supplementary.pdf]

Table S1. Direct antimicrobial activity of the 35 isolates from European hakes against pathogenic bacteria by a Stab-On-Agar Test (SOAT)[a].

Isolates	*Lc. garvieae* CF00021	*Lc. garvieae* CLG4	*L. monocytogenes* CECT911	*L. ivanovii* CECT913	*Y. ruckeri* LMG3279	*A. hydrophila* CECT839	*A. hydrophila* CECT5734	*A salmonicida* CLFP-23	*A. salmonicida* CECT4237	*Ls. anguillarum* CECT4344	*T. maritimum* NCIMB2154	*T. maritimum* CECT1161	*E. tarda* CECT886	*St. parauberis* LMG22252
Lc. garvieae MAH1	+	++	-	-	++	-	-	+	-	++	+	-	+	+
Lc. garvieae MAH3	+	+	-	-	++	-	-	+	+	++	+	-	-	+
Lc. garvieae MAH6	-	+	-	-	-	-	-	++	++	+	+	-	+	+
Lc. garvieae MAH7	-	-	-	-	++	-	-	+	+	++	++	++	+	++
Lc. garvieae MAH10	+	+	-	-	++	-	-	+	+	++	++	++	-	+
Lc. garvieae MAH13	+	+	-	-	++	-	-	+	-	+++	++	+	-	-
Lc. garvieae MAH16	++	-	-	-	-	-	-	+	-	+	+	-	-	-
Lc. garvieae MAH17	++	+	-	-	-	-	-	+	-	++	+	-	-	+
Lc. garvieae MAH18	++	+	-	-	-	-	-	+	+	++	+	-	+	-
Lc. garvieae MAH20	+	-	++	-	-	-	-	+	-	++	+	+	+	+
Lc. garvieae MBI13	++	+	-	-	-	+++	+	++	++	-	+	-	-	-
Lc. garvieae MBI14	+	+	+	-	-	-	-	++	++	-	+	-	-	-
Lc. garvieae MBI17	+	+	+	++	-	++	-	++	+++	-	+	-	-	++
Lc. garvieae MBI18	+	++	+	-	-	++	-	+	++	-	+	-	-	++
Lc. garvieae MBH4	-	-	-	++	-	+	+	+	++	-	++	++	-	-
Lc. garvieae MBH5	+	-	-	+	-	++	++	+	++	-	+	-	-	+
Lc. garvieae MBH6	-	-	-	-	-	+	+++	+	+	-	-	-	-	++
Lc. garvieae MBH14	-	-	-	-	-	+	+++	-	++	-	+	-	-	++
Lc. garvieae MBH17	-	++	-	-	-	+	+	-	++	-	++	+	-	++
Lc. garvieae MBH20	-	-	-	-	-	-	+++	-	+++	-	+	-	-	++
Lc. garvieae MFI1	+	+	+	-	-	++	-	++	+++	++	-	-	++	++
St. salivarius MDI13	++	+	-	-	++	+	++	+	+	++	-	-	-	++
St. salivarius MDI14	-	-	-	-	-	++	-	++	+	+++	-	-	-	-
St. salivarius MDI17	-	-	-	-	++	++	++	+	+	-	-	-	-	-
St. salivarius MDI18	-	-	-	-	++	++	++	-	-	+	-	-	-	++
St. salivarius MDI19	-	-	-	-	-	+	++	++	+	+	-	-	-	+++
St. salivarius MDI20	-	-	-	-	-	+	-	++	-	+	-	-	-	-
E. avium MEI4	-	-	-	-	-	-	-	-	-	-	-	++	+	++
E. avium MEI19	-	-	-	-	-	-	-	-	+	-	-	+	+	-
E. avium MEI29	-	-	-	-	-	+	+	-	+	-	-	++	-	++
E. avium MGH6	-	-	-	-	+	-	++	-	-	-	-	-	++	+
E. avium MHH18	-	-	+	-	+	+	+	++	+	++	+	++	-	+++
E. avium MHH19	-	-	+	-	-	+	+	++	++	++	++	++	-	-
Lb. sakei MEI5	+	+	+	-	-	+	++	+	++	-	+	++	++	++
Lt. carnosum MHI5	+	+	+	-	-	+	-	++	++	++	-	+	-	+

[a]Scores reflects growth inhibition ranges (inhibition zones diameters in mm): -, no inhibition; +, 3-5 mm; ++, 5-10 mm; +++, 10-15 mm.
